# Association between CYP2D6 genotype and treatment effectiveness and safety in 99 hospitalized patients with major depressive disorder a retrospective cohort study CYP2D6 genotype and antidepressant response

**DOI:** 10.1038/s41397-026-00428-y

**Published:** 2026-08-19

**Authors:** Aleksandra Petković Ćurčin, Aleksandra Jeremić, Danilo Joković, Gordana Šupić, Katarina Simić, Filip Milosavljević, Zvezdana Stojanović, Marin M. Jukić

**Affiliations:** 1 Faculty of Medicine, Military Medical Academy, University of Defense, 11040 Belgrade, Serbia; 2 Institute for Medical Research, Military Medical Academy, 11040 Belgrade, Serbia; 3 Faculty of Pharmacy, University of Belgrade, 11221 Belgrade, Serbia; 4 Clinic for Psychiatry, Military Medical Academy, 11040 Belgrade, Serbia; 5 Department of Psychiatry, Berkshire Medical Center, 01201 Pittsfield, MA United States of America; 6 Department of Psychiatry and Psychotherapy, School of Medicine, Technische Universität München, 81675 München, Germany; 7 Department of Physiology and Pharmacology, Karolinska Institute, 17177 Solna, Sweden

**Keywords:** Depression, Risk factors, Genotype

## Abstract

Cytochrome P450 2D6 (CYP2D6) is a polymorphic enzyme that influences antidepressant metabolism. This retrospective cohort study investigated the association between *CYP2D6* genotype and treatment outcomes in 99 hospitalized patients with major depressive disorder (MDD) in Belgrade, Serbia. Patients were classified as poor (PM, *n* = 5), intermediate (IM, *n* = 21), or normal metabolizers (NM, *n* = 73). Effectiveness and tolerability were assessed from admission to discharge (~4 weeks). Hamilton Depression Rating Scale (HAM-D) score reduction was the primary outcome; tolerability was measured using the Toronto Side Effects Scale (TSES). Compared with NMs, HAM-D score reductions were 4.3 and 9.0 points lower in IMs and PMs. TSES scores were 1.3 and 2.3 points higher in IMs and PMs, respectively. Central nervous system and gastrointestinal effects were more frequent in IMs and PMs; sexual dysfunction did not differ. Reduced CYP2D6 activity was associated with poorer outcomes, indicating the potential usefulness of *CYP2D6* genotyping in MDD.

## Introduction

Depressive disorders lead to the disability of more than 280 million individuals [1, 2], while two thirds of suicide victims are depressed at the time of their death [3]. Numerous major depressive disorder (MDD) patients experience treatment failure due to lack of effectiveness or intolerable side effects [4–6]. Since the development of new antidepressant drugs is very slow, there is an urgent need for personalization of treatment with currently available antidepressants, for example, by utilization of pharmacogenomics information in therapeutic decision making protocols [7]. Despite the evidence that pharmacogenomics-guided approach improves antidepressant treatment outcomes [8–12], the effect sizes are still not sufficiently convincing and they vary among sources due to inconsistencies in cohorts analyzed and study settings. Consequently, further clinical research focused on specific gene-drug interactions and adequate characterization of clinically impactful and actionable pharmacogenetics associations is needed to facilitate the implementation of meaningful pharmacogenetics testing into clinical practice.

Cytochrome P450 2D6 (CYP2D6) and 2C19 (CYP2C19) are polymorphic enzymes that metabolizes the majority of commonly prescribed antidepressants [13, 14]. Functional genetic variants of *CYP2D6* and *CYP2C19* are highly prevalent and they cause the significant inter-individual variability in enzymatic capacity. Patients are usually categorized as poor metabolizers (PMs), intermediate metabolizers (IMs), normal (or extensive) metabolizers (NMs) and ultra-rapid metabolizers (UMs) based on *CYP2C19* and *CYP2D6* genotype, with the rapid metabolizer (RM) phenotype additionally defined for *CYP2C19* [15, 16]. PMs are at increased risk of elevated drug exposure and intolerable adverse effects, whereas IMs generally require careful dose selection or monitoring for certain antidepressants. In contrast, UMs are at higher risk of subtherapeutic drug levels and inadequate treatment response. As both mentioned risks affect antidepressant treatment successfulness [15, 17, 18], the knowledge of *CYP2C19* and *CYP2D6* genotype can potentially be useful for clinicians in helping them with selection of appropriate antidepressant drugs and their dosage. Our previous work showed that CYP2C19 slow metabolizer phenotype is associated with lower antidepressant efficacy and tolerability [19]. Related to the association between *CYP2D6* genotype and the effectiveness and tolerability of antidepressants that has been extensively studied, results remain inconsistent [20]. Many studies advocate the importance of matching the choice of antidepressant with *CYP2D6* metabolizer status, while the CYP2D6 enzymatic capacity, as determined by genetic variation, has been associated with differences in antidepressant effectiveness [21–23] and tolerability [22, 24, 25]. Next, *CYP2D6* PMs were at five times higher risk to switch their antidepressants [26–28] or discontinue treatment compared to NMs [29, 30]. However, there are many other adequately powered studies that failed to observe significant association between *CYP2D6* phenotype and antidepressant treatment effectiveness [31–33] or tolerability [32, 34].

Currently, guidelines published by various expert groups, such as the Clinical Pharmacogenetics Implementation Consortium (CPIC), the Dutch Pharmacogenetics Working Group (DPWG), and the US Food and Drug Administration (FDA) provide the instructions to clinicians on how to use *CYP2D6* genotype to personalize antidepressant treatment. However, discrepancies between these recommendations are still apparent, underlining the lack of certainty about how *CYP2D6* genotype needs to be used to maximize antidepressant treatment successfulness [17, 35–37]. This naturalistic retrospective cohort study aimed to investigate the association of *CYP2D6* genotype with antidepressant treatment effectiveness and safety in patients hospitalized due to major depressive disorder.

## Material and methods

This naturalistic hospital-based retrospective cohort study was conducted between May 2016 and January 2018 at the Department of Psychiatry, Military Medical Academy (Belgrade, Serbia). This study complied with the principles of the Declaration of Helsinki (2013). The protocol was approved by the Ethics Committee of the Military Medical Academy in Belgrade on March 29, 2016, and all participants signed written informed consent before being subjected.

### Study participants and exposures

Patients were eligible for inclusion if they had been (1) diagnosed with MDD, (2) receiving stable treatment with one or two antidepressants, and (3) hospitalized in a tertiary care facility due to the severity of their depression symptoms. All participants were members of a military cohort (active-duty personnel or veterans) or their family members. The diagnosis of major depressive disorder was established according to ICD-10 criteria. MINI 5.0.0. (Mini International Neuropsychiatric Interview) [38] was used as a screening tool; exclusion criteria included (1) age under 18 or over 65 years, (2) a Mini Mental State Examination (MMSE) score below 24 at enrollment, (3) less than eight years of formal education, and (4) a diagnosis of mental retardation according to ICD-10 criteria. Psychiatric comorbidities were excluded based on MINI results, while psychotic features were permitted only when defined as part of a severe depressive episode. Treatment choice and dosing were unrestricted and determined by the treating psychiatrists. The treatment recorded in the database corresponds to the therapy at discharge, after any adjustments or additions made during hospitalization. To enable dose comparisons, antidepressant doses were converted to fluoxetine equivalents [39].

Patients were clinically assessed at three predefined time points: (1) At the beginning of the study (visit 0, V0) after hospital admission, (2) during interim assessment (visit 1, V1), two weeks after hospitalization, and (3) during final assessment (Visit 2, V2) on discharge from hospital (4–6 weeks after admission). *CYP2D6* genotyping and categorization into metabolizer groups were performed retrospectively after study completion. Consequently, clinicians and investigators assessing psychometric outcomes were blinded to *CYP2D6* genotype, and treatment decisions were not guided by pharmacogenetics data.

The *CYP2C19* data from the previous study [19] were not available to the treating psychiatrists during the trial. This report includes these newly obtained pharmacogenetics data from stored DNA samples, under approved ethical procedures.

### *CYP2D6* genotyping

Peripheral blood samples were collected in EDTA tubes and stored at −20 °C until DNA isolation using the PureLink Genomic DNA Mini Kit (Invitrogen, USA). CYP2D6 genotyping was performed with the commercially available TaqMan SNP Genotyping Assays (Applied Biosystems) on a 7500 Real-Time PCR System (Applied Biosystems, Foster City, CA, USA) using the allele discrimination method to genotype the polymorphisms. Detailed genotyping procedures, assayed variants, and copy number analysis are provided in the Supplementary Material.

Patients were categorized based on genotype-predicted enzymatic activity. CYP2D6 metabolizer status was defined according to CPIC/DPWG consensus recommendations [40] using standard genotype-to-phenotype translation. Patients were classified as PM, IM and NM. No UM patients were identified in this cohort. An additional exploratory analysis using an alternative classification approach [41] was performed, with results presented in the Supplementary Material.

### Clinical measurements and study outcomes

At V0, structured questionnaires were administered to collect the data on (1) socio-demographic status, (2) medical history of depression, (3) prescribed antidepressant regimens and dosages, (4) substance abuse history, and (5) concurrent medications. MMSE scores were recorded at each visit to monitor potential cognitive impairment. Depression symptom severity was evaluated by psychiatrists using the 21-item Hamilton Depression Rating Scale (HAMD) [42] complemented with Clinical Global Impression (CGI) scales for disease severity (CGI-S) and treatment improvement (CGI-I) [43]. Additionally, the 21-item Beck Depression Inventory-IA [44] was utilized as a patient self-reported measure of treatment response. Assessments were performed at all visits, except in case of CGI-I score, which was not applicable at V0. Treatment effectiveness was quantified by a decrease in symptom severity from baseline measured by HAMD and BDI-IA, with the reduction of HAMD score as the primary outcome. Next, response incidence analyzed separately as a categorical outcome and was measured as the proportion of patients with a ≥ 50% reduction in HAMD and BDI-IA scores. Finally, absolute CGI-S and CGI-I scores at V1 and V2 were compared across metabolizer groups.

Treatment tolerability was assessed at V1 and V2 using the Toronto Side-Effects Scale (TSES) [45] complemented with the Clinical Global Impression - Efficacy Index (CGI-E) [43]. The TSES evaluated the frequency and severity of adverse effects categorized into three domains: central nervous system (CNS) effects, gastrointestinal (GI) disturbances, and sexual dysfunction (SF). Composite intensity scores were calculated by multiplying frequency and severity ratings and average intensity scores were compared between groups for overall side-effect burden and for each domain separately [45]. Additionally, CGI-E was calculated at V1 and V2 as the ratio of clinical benefit to side-effect burden and was used as a secondary outcome of treatment tolerability.

### Statistical data analysis

The statistical analyses were performed using SPSS Statistics 20 software (IBM, USA). This sample size was calculated to detect a difference of three HAMD points, which is considered the minimal clinically important improvement [46] with a statistical power of 85% [47]. Normality of the data was assessed by Shapiro-Wilk test. Socio-demographic and medical history data were compared using one-way ANOVA (normally distributed data) or by the Kruskal-Wallis. Fisher’s exact test was used for comparison of categorical variables. Absolute HAMD and BDI-IA values were compared using repeated-measures ANOVA to assess changes between visits. Between-group comparisons of HAMD at each visit, as well as relative score changes, were further assessed using analysis of covariance (ANCOVA), with Tukey post-hoc test for between-group comparison. Additionally HAMD and BDI-IA response rates were evaluated using the binary logistic regression. Covariates in both models were the age, gender, baseline diagnosis, episode duration, and fluoxetine-equivalent dose because all these factors have been shown to influence the effectiveness of antidepressant treatment [48, 49]. TSES intensity scores, MMSE scores, and CGI scores were compared between groups using the Kruskal-Wallis test, with Mann-Whitney U tests for *post hoc* comparisons. In addition to the primary analysis focusing on CYP2D6, combined analyses considering both CYP2D6 and CYP2C19 metabolizer status were performed using the same statistical methods.

Sensitivity analyses were performed to assess the robustness of the findings by excluding six patients receiving non-CYP2D6 enzyme substrate antidepressants [14] and by applying an alternative CYP2D6 classification approach [41] (Supplementary Material). Additionally, patients were reclassified according to phenoconversion-adjusted CYP2D6 phenotypes based on treatment at V2 when CYP2D6 inhibitors were co-administered with CYP2D6 substrates; fluoxetine monotherapy was not considered phenoconversion [50]. FDR correction of *p*-values was applied whenever multiple comparisons were performed, all tests were two-sided, and corrected *p*-values lower than 0.05 were interpreted as statistically significant difference.

## Results

Study results were reported in accordance with STROBE statement guidelines for cohort studies (Table S1) [51].

### Patient baseline characteristics

Of the 115 eligible patients, 102 patients agreed to participate in the study and completed the protocol, while three patients were excluded due to inconclusive *CYP2D6* genotyping. Based on *CYP2D6* genotype, patients were categorized into 73 NMs, 21 IMs, and 5 PMs. All patients were Caucasians. No significant differences were observed in demographic variables and substance abuse. PMs had a longer history of depression than both NMs and IMs—by 8 and 10 years, respectively. IMs had longer hospitalization compared to NMs by 9 days. At admission, the median HAMD and BDI-IA scores were 33 and 35, respectively. IMs showed higher median HAMD (34 vs. 32) and BDI-IA (46 vs. 34) scores than NMs. The most frequently prescribed antidepressants were mirtazapine (34), escitalopram (22), trazodone (20), mianserin (14), venlafaxine (12), sertraline (11). Among these medications, those considered CYP2D6 substrates are listed in Supplement (Table S2). All baseline values of the study cohort and study groups are shown in detail in Table 1.

**Table 1:** Population characteristics.

Demographics	Total	NM	IM	PM	Statistics
	*n* = 99	*n* = 73	*n* = 21	*n* = 5	
Sex *(Male/Female)*	56/43	41/32	13/8	2/3	*p* > *0.1*
Age *(years)*	47 (11)	46 (11)	49 (12)	50 (7.3)	*p* > *0.1*
Education *(Elementary/Highschool/University)*	11/58/30	8/41/24	2/13/6	1/4/0	*p* > *0.1*
Employment *(Unemployed/Employed/Retired)*	37/40/22	27/32/14	7/7/7	3/1/1	*p* > *0.1*
Marital status *(Married/Unmarried)*	60/39	42/31	13/8	5/0	*p* > *0.1*
**Substance abuse**					
Alcohol *(nof users)*	27	24	3	0	*p* = *0.09*
Smoking *(nof users)*	45	32	11	2	*p* > *0.1*
Psychoactive substances *(nof users)*	0	0	0	0	*p* > *0.1*
**Depression medical history**					
Duration of depression*(Years)*	5.0(1.0 - 10)	5.0(1.0 – 10)^b^	7.0(2.0 – 12)^b^	15(12 – 23)^a^	***p*** = ***0.011***
Duration of current episode*(Months)*	2.0(1.0–3.0)	2.0(1.0–3.0)	2.0(1.0–6.0)	3.0(2.0–7.5)	*p* > *0.1*
First time hospitalized*(n)*	41	32	9	0	*p* = *0.06*
Recurrent MDD (n, %)	53 (54%)	36 (49%)	14 (67%)	3 (60%)	*p* > *0.1*
Duration of hospitalization*(days)*	24(21–32)	23(21–30)^a^	32(22–41)^b^	32(26–52)^a, b^	***p*** = ***0.018***
Antidepressant monotherapy	54 (54%)	40 (55%)	11 (52%)	3 (60%)	*p* > *0.1*
Total antidepressant dose (*Fluoxetine equivalent mg*)	32(19–44)	32(17–41)	26(18–45)	40(19–51)	*p* > *0.1*
CYP2D6 substrate use (n, %)	93 (94%)	68 (93%)	20 (95%)	5 (100%)	*p* > *0.1*
HAMD score at admission	33(28–35)	32(27–34) ^a^	34(33–39) ^b^	27(30–40) ^a, b^	***p*** = ***0.008***
BDI-IA score at admission	35(30–45)	34(28–40)^a^	46(37–52)^b^	37 (30–48)^a,b^	***p*** = ***0.003***
Depression diagnosis *(Mild or Moderate/Severe/Severe with psychosis)*	14/65/20	12/50/11	2/12/7	0/3/2	*p* > *0.1*
***CYP2D6*** **genotype**		*1/*1, n = *58* *1/*2, n = *6* *1/*9, n = *2* *1/*41, n = *6* *2/*41, n = *1*	*1/*3, n = *2* *1/*4, n = *12* *1/*6, n = *2* *1/*5, n = *3* *9/*5, n = *1* *4/*41, n = *1*	*4/*4, n = *4* *3/*3, n = *1*	

Data presented as mean (standard deviation), as median (IQR), or as absolute numbers. Different letters in a superscript annotate groups with significant between-group differences.

*NM* Normal metabolizers, *IM* Intermediate metabolizers, *PM* Poor metabolizers, *HAMD* hamilton depression rating scale, *BDI-IA* Beck depression inventory.

### Association between CYP2D6 genotype and treatment effectiveness and tolerability

**Fig. 1:**
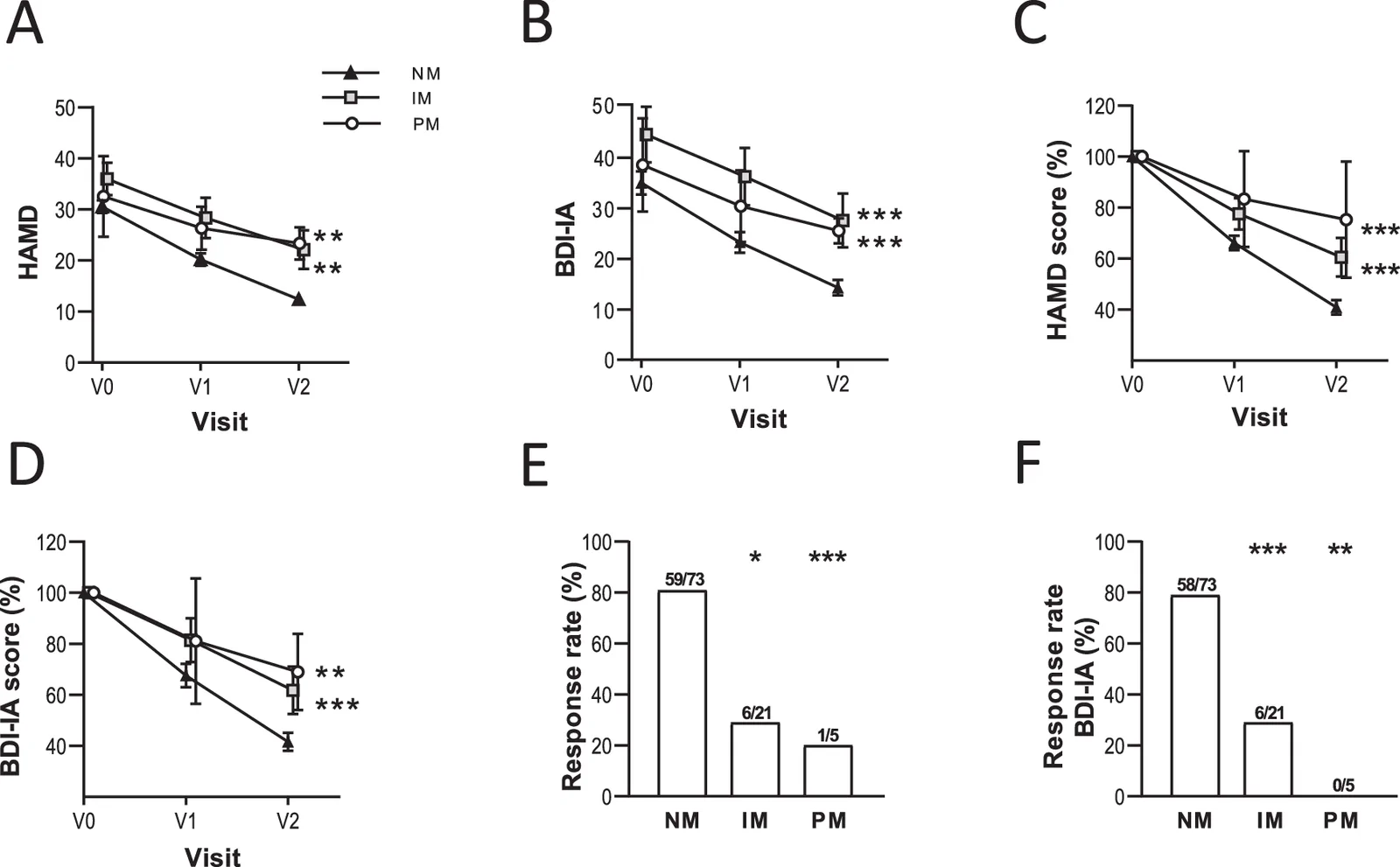
Association between antidepressant treatment effectiveness and *CYP2D6* genotype. **A** At V2, PMs showed a 9.0 points smaller absolute HAMD decrease compared to NMs [95% CI: 2.8–15, *p* = 0.002], while IMs had a 4.3 points smaller decrease [95% CI: 1.0–7.7, *p* = 0.006]. **B** A repeated measures ANOVA revealed a significant main effect of visit on BDI-IA scores (*p* < 0.001), indicating a general reduction in symptoms over time. Additionally, a significant main effect of metabolizer group was observed (*p* < 0.001), indicating that average BDI-IA scores differed between CYP2D6 metabolizer category. **C** At V2, PMs had a 32% [17–48%, *p* < 0.001] lower absolute decrease in HAMD scores compared to NMs, while IMs showed a 19% [11–28%, *p* < 0.001] lower decrease. **D** At V2, IMs had a 19% [9–29%, *p* < 0.001] smaller decrease in BDI-IA scores than NMs, while PMs had a 26% [7–44%, *p* = 0.004] smaller decrease. **E** Response rate was defined as proportion of patients with HAMD score reduction of 50% or higher from V0 to V2. IMs and PMs had 52% (*p* = 0.024) and 61% (*p* < 0.001) lower response rate, compared with NMs, respectively; (**F**) Response rate was defined as proportion of patients with ≥50% reduction in BDI-IA score from V0 to V2. IMs and PMs had 42% (*p* < 0.001) and 81% (*p* = 0.001) lower response rates compared with NMs, respectively.Covariates in ANCOVA models and logistic regression included age, sex, diagnosis at V0, episode duration, and fluoxetine-equivalent dose. HAMD Hamilton Depression Rating Scale, BDI-IA Beck depression inventory, NM Normal CYP2D6 metabolizer, IM Intermediate metabolizers, PM Poor CYP2D6 metabolizer, V0 hospital admission, V1 2 weeks after the admission, V2: 4–6 weeks after the hospital admission.*p < 0.05, ** p < 0.01, *** p < *0.001, whiskers represent CI 95%*.

To evaluate whether treatment effectiveness was associated with *CYP2D6* genotype, we compared the reduction in depression symptom severity across *CYP2D6* genotype-defined metabolizer groups. Compared to NMs, IMs and PMs exhibited 4.3 and 9.0 points lower HAM-D score reductions (Fig. 1A) and higher CGI-I scores at V2 (Table 2), reflecting less clinician-reported improvement. Similarly, BDI-IA scores improved over time and differed between the groups at both follow-up visits (Fig. 1B). In terms of relative symptom reduction, HAMD score decrease was lower by 19 and 32% in IMs and PMs compared to NMs, respectively (Fig. 1C), while BDI-IA scores decrease was lower by 19 and 26% in IMs and PMs, respectively (Fig. 1D). Based on HAMD, response rates were lower by 52 and 61% in IMs and PMs compared with NMs, respectively (Fig. 1E). Based on BDI-IA, response rates were lower by 50 and 79% in IMs and PMs compared with NMs, respectively (Fig. 1F). Altogether, treatment effectiveness was substantially lower in CYP2D6 intermediate and poor metabolizers, compared with normal metabolizers.

**Table 2:** MMSE and CGI scores in patients with different *CYP2D6* metabolizer status.

Clinical scale		NM	IM	PM
		*n* = 73	*n* = 21	*n* = 5
	V0	30(28–30)	29(27–30) *p* = *0.079*	28(25.5–29.5) *p* = *0.095*
**MMSE**	V1	30(29–30)	29(27–30) ***p*** = ***0.013***	29(26–30) *p* = *0.158*
	V2	30(30–30)	30(28–30) ***p*** = ***0.003***	29(26–30) *p* = *0.083*
	V0	5(5–6)	6(5.5–6) ***p*** = ***0.007***	6(5.5–6.5) *p* > *0.1*
**CGI-S**	V1	4(4–5)	5(4–5) ***p*** = ***0.05***	5(4.5–6) *p* = *0.056*
	V2	3(3–4)	4(3.5–4.5) ***p*** = ***0.008***	4(3–5) *p* > *0.1*
	V0	7(6–7)	7(6–7) *p* > *0.1*	7(6.5–7) *p* > *0.1*
**CGI-I**	V1	2(2–2)	2(2–3) ***p*** < ***0.0001***	3(2–5) ***p*** = ***0.041***
	V2	2(1–2)	2(2–2.5) ***p*** < ***0.0001***	2(2–4) ***p*** = ***0.013***
**CGI-E**	V1	3(3–3)	2(2–3) ***p*** < ***0.0001***	1(1–2.2) ***p*** = ***0.004***
	V2	3(3–4)	3(1.5–3) ***p*** < ***0.0001***	1.5(1.2–2.5) ***p*** = ***0.001***

*p*-values represent below IM and PM values represent statistical comparison with NMs. Data is shown as median (IQR).

MMSE mini mental state examination, CGI-S clinical global impression scale for severity of the illness, CGI-I clinical global impression scale for the patient’s improvement, CGI-E clinical global impression scale: efficacy index;V0 - Hospital admission; V1 - 2 weeks after the admission; V2: 4–6 weeks after the hospital admission, NM normal CYP2D6 metabolizer, IM intermediate CYP2D6 metabolizer, PM poor CYP2D6 metabolizer.

**Fig. 2:**
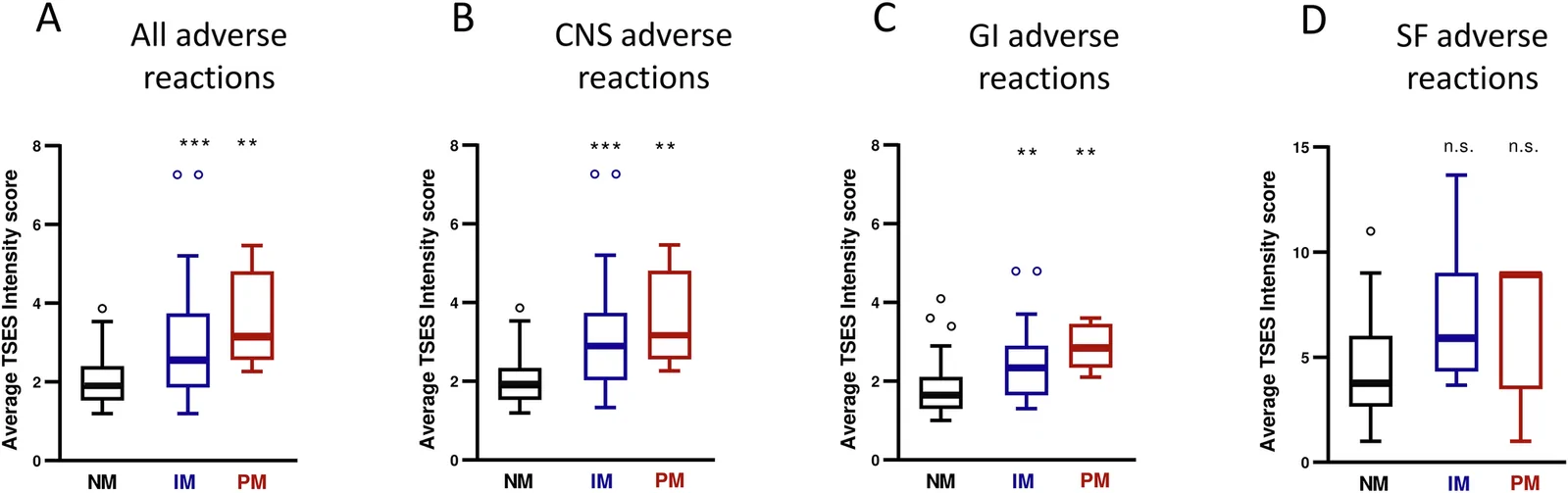
Association between antidepressant treatment tolerability and *CYP2D6* genotype. **A** At V2, median intensity scores for all adverse reactions combined were significantly higher in both IMs: 3.6 [IQR:2.9–4.1, *p* < 0.001]) and PM: 4.6 [IQR: 3.3–5.3, *p* = 0.002], compared with NMs: 2.3 [IQR: 1.8–3.1]. **B** Median intensity scores for CNS-related adverse reactions were also higher in both IMs: 2.9 [IQR: 2.0–3.7, *p* < 0.001] and PMs: 3.2 [IQR: 2.6–4.8, *p* = 0.002], compared to NMs: 1.9 [IQR: 1.5–2.3]. **C** Similarly, both IMs: 2.3 [IQR: 1.6–2.9, *p* = 0.004] and PMs: 2.9 [IQR: 2.4–3.4, *p* = 0.001] exhibited higher median intensity scores for GI-related adverse reactions compared to NMs: 1.6 [IQR: 1.3–2.1]. **D** However, for SF-related adverse reactions, differences between NMs: 3.7 [IQR: 2.7–6.0], IMs: 6.0 [IQR: 3.8–9.0], and PMs: 9.0 [IQR: 3.5–9.0] did not reach statistical significance (*p* = 0.082) *CNS central nervous system, GI gastrointestinal, SF sexual function, TSES Toronto Side-Effects Scale, NM Normal CYP2D6 metabolizer, IM Intermediate CYP2D6 metabolizer, PM Poor CYP2D6 metabolizer, V2: 4–6 weeks after the hospital admission*, IQR interquartile range.*p < 0.05, ** p < 0.01, *** p < *0.001, Boxes represent interquartile range, whiskers represent minimum and maximum and dots represent outliers*.

To evaluate whether antidepressant treatment tolerability was associated with *CYP2D6* genotype, we compared the severity of adverse drug reactions (ADRs) between *CYP2D6* genotype-defined subgroups. At V2, IMs and PMs exhibited 1.3 and 2.3 higher median TSES intensity scores compared to NMs, respectively (Fig. 2A). For CNS-related ADRs (Fig. 2B), IMs and PMs showed 1.0 and 1.3 higher intensity scores compared with NMs, respectively. For GI-related ADRs (Fig. 2C), IMs and PMs exhibited 0.7 and 1.3 higher median intensity scores compared with NMs, respectively, whereas intensity of SF-related adverse drug reactions were not different between *CYP2D6* metabolizer groups (Fig. 2D). Additionally, compared with NMs, IMs and PMs exhibited significantly lower effectiveness-to-tolerability ratios at V2, as measured by CGI-E scores (Table 2), indicating worse overall treatment tolerability. Altogether, treatment tolerability was worse in *CYP2D6* intermediate and poor metabolizer groups compared with normal metabolizers.

### Association between combined CYP2D6–CYP2C19 metabolizer status and treatment effectiveness and tolerability

To complement the CYP2D6 analysis, we evaluated combined CYP2D6–CYP2C19 metabolizer status (phenotype frequencies in Table S5). In ANCOVA including both enzymes, CYP2D6 (*p* = *0.006*) and CYP2C19 (*p* = *0.001*) remained independent predictors of absolute HAMD reduction, with a modest interaction effect (*p* = *0.018*; η² = 0.12).

Treatment effectiveness declined progressively compared with the reference group (CYP2D6 normal + CYP2C19 normal/rapid): patients with reduced function of one enzyme showed a 4.0-point lower HAMD reduction (17% relative), while those with reduced function of both enzymes showed a 7.7-point lower HAMD reduction (30% relative). A similar pattern was observed for BDI-IA scores (Table S6).

Tolerability outcomes followed the same trend: median TSES intensity increased from 2.3 in the reference group to 3.1 and 3.6 in the one- and two-enzyme impairment groups, with consistent increases in CNS- and GI-related ADRs. Overall, the combined analysis demonstrates a cumulative effect of reduced CYP2D6 and CYP2C19 activity on both treatment effectiveness and tolerability (Table S6).

### Sensitivity analysis

>Decision to exclude patients not treated with CYP2D6 substrate drugs did not meaningfully change the magnitudes or the significance levels of the between-group changes in the treatment effectiveness and tolerability readouts. Similarly, repeating the analyses using the alternative CYP2D6 classification approach and reclassifying patients according to phenoconversion-adjusted phenotypes produced no meaningful changes. Full details of all sensitivity analyses are provided in the Supplement (Tables S3–S4, S7–S8; Figs. S1–S6).

## Discussion

The results indicate that slower *CYP2D6* metabolism in this cohort of hospitalized patients suffering from MDD contributed to worse antidepressant treatment outcomes compared with normal *CYP2D6* metabolizers. However, the small number of CYP2D6 poor metabolizers (*n* = 5) limits statistical power and consequently, findings related to this subgroup should be interpreted with caution.

Worse effectiveness was observed in assessment by both clinician-rated (HAMD, CGI-I, CGI-S) and patient-rated (BDI-IA) psychometry assessment tools, while worse tolerability was observed in both TSES scores and CGI-E effectiveness/tolerability ratios. These results are concordant with previously published reports indicating worse effectiveness [21, 22, 52] and tolerability [22, 24, 25] of antidepressant treatment in patients with genetically-encoded lower *CYP2D6* enzymatic capacity. However, these results are in contrast with other reports that failed to observe association between *CYP2D6* genotype and antidepressant treatment outcomes [31–34].

Although PMs had higher doses and longer illness duration, which could potentially contribute to poorer outcomes, ANCOVA models including dose as a covariate indicated that dosing differences did not explain the higher incidence of ADRs. Importantly, *CYP2D6* genotyping was performed retrospectively and was not available to treating clinicians; therefore, dose adjustments were based on clinical response rather than pharmacogenetics data. Therefore, the higher doses observed in some patients likely reflect attempts to manage insufficient treatment response rather than genotype-guided prescribing. Moreover, a supplementary composite analysis incorporating both *CYP2D6* and *CYP2C19* genotypes demonstrated that patients with reduced function of both enzymes exhibited the poorest treatment outcomes and the highest overall burden of adverse drug reactions.

The obtained results suggest a potential association between CYP2D6 and CYP2C19 metabolic capacity and treatment outcomes, indicating that genotype information may be useful for clinicians when considering drug, dosage, and treatment strategies. Indeed, well replicated results indicate that PMs are at increased risk of in tolerable adverse drug reactions even under standard dosing, which often leads to drug discontinuation before the benefits can be achieved [22, 24, 25]. Consequently, dose adjustments or alternative medications in *CYP2D6* PMs are recommended across relevant clinical guidelines for numerous antidepressant drugs [15, 36]. However, most of the guidelines focus only on PMs and overlook the significance of IMs, despite the evident differences in antidepressant blood levels between IMs and NMs [53]. While the clinical significance of being an IM is likely subtler than being a PM, according to this and other studies [54], IMs also require different treatment and dosing strategies, especially when the drug has a narrow therapeutic window [23, 55].

In addition, one less likely, but plausible explanation for the observed results concerns the considerable presence of *CYP2D6* in the brain, which means that local metabolism of antidepressants may be affected by *CYP2D6* genotype [56]. Reduced *CYP2D6* activity may be associated with altered intracerebral levels of antidepressants or their metabolites independent of blood levels, potentially due to different effects on neurotransmitter homeostasis in the brain. Although formal dosing recommendations based on *CYP2D6* genotype exist for only a subset of antidepressants, CYP2D6 contributes to the metabolism of most drugs used in this study, which may partly explain the observed influence of CYP2D6 metabolizer status and the role of active metabolites on treatment outcomes. However, future studies incorporating therapeutic drug monitoring of both parent compounds and metabolites are needed to clarify these mechanisms. In conclusion, the observed trend between slower CYP2D6 and CYP2C19 metabolism (both independently and combined) and poorer treatment outcomes suggests the potential benefits of using preemptive *CYP2C19/CYP2D6* genotyping as part of the standard treatment routine. Although the small number of PMs limits the strength of this conclusion, their low prevalence is consistent with previous reports in the European population [57]. In theory, the ability to identify PMs and IMs prior to initiating treatment could provide clinicians with useful information to better individualize therapy, reduce adverse effects, and improve the successfulness of antidepressant therapy. These results are particularly useful when considering hospitalized patients suffering from depression, like the cohort analyzed here, where there is less margin for error and more at stake in case of suboptimal treatment. Importantly however, studies with prospective design, preemptive *CYP2D6* and *CYP2C19* genotyping, and examination of pharmacokinetic parameters are still needed to unequivocally demonstrate the usefulness of knowing *CYP2D6* and *CYP2C19* genotype in terms of improving antidepressant treatment successfulness. These findings should be interpreted cautiously and require confirmation in larger, prospective studies incorporating pharmacokinetic measurements.

### Limitations

The main limitation is lack of availability of pharmacokinetic data meaning that it was not possible to directly link *CYP2D6* genotype to drug concentration in the blood. Therapeutic drug monitoring, a useful tool in neuropsychopharmacology [14], was not available, and data on liver/kidney function, body weight, and comorbidities were missing, which may have influenced metabolism. The CYP2D6 panel was limited to major European alleles [57], possibly causing occasional phenotype misclassification, particularly among IMs. Next, due to the small number of PMs, all statistical comparisons related to this category should be interpreted with caution. Concomitant use of multiple antidepressants, common in clinical practice, further complicates interpretation of results due to interference between *CYP2D6* gene–drug and drug-drug interactions. Non-antidepressant concomitant medications were not assessed, which may limit the accurate assessment of phenoconversion and introduce residual confounding, as phenoconversion due to other drugs could not be evaluated. In addition, phenoconversion was assessed based on treatment at the time of outcome evaluation and does not account for dynamic changes in medication during hospitalization. Treatment-resistant depression (TRD) status was not systematically assessed. Finally, numerous factors limit the generalizability of the results obtained to general population because: (**1**) the cohort did not include UMs, (**2**) the cohort consisted primarily of military personnel and consequently the sample was predominantly male (**3**) all patients had relatively severe depression that required hospitalization, (**4**) the cohort included only Caucasian participants.

## Supplementary information

SUPPLEMENTAL MATERIAL

## Data Availability

The datasets generated during and/or analysed during the current study are available from the corresponding author on reasonable request.
