# Supplementary material for: Association between CYP2D6 genotype and treatment effectiveness and safety in 99 hospitalized patients with major depressive disorder a retrospective cohort study CYP2D6 genotype and antidepressant response
Source: Pharmacogenomics J. 2026 Aug 19;26(5):34. doi: 10.1038/s41397-026-00428-y (PMC13489959; doi:10.1038/s41397-026-00428-y)
Supplement: Supplementary file 1 [file 41397_2026_428_MOESM1_ESM.docx]

**Association between CYP2D6 genotype and treatment effectiveness and safety in 99 hospitalized patients with major depressive disorder: a retrospective cohort study**

**SUPPLEMENTARY MATERIAL**

**Text S1: Supplementary Methods: *CYP2D6* genotyping**

Patients were genotyped for common non-functional alleles (*CYP2D6Nonf*)*,* including *CYP2D6*3* (rs35742686), *CYP2D6*4* (rs3892097), *CYP2D6*6* (rs5030655) and for common alleles associated with decreased enzymatic capacity (*CYP2D6Decr*), including *CYP2D6*9* (rs5030656), *CYP2D6*10* (rs1065852) and *CYP2D6*41* (rs28371725) alleles. The *CYP2D6*2* allele was genotyped based on presence of rs16947 variant. Rare alleles in this population, such as *CYP2D6*17* (<0.5%) [40], were not included in the analysis. Gene copy number variation was assessed to detect deletions or multiplications of entire genes according to the manufacturer’s protocol (Assay ID: Hs00010001_cn; Thermo Fisher Scientific, Waltham, MA). This assay targets exon 9 of the *CYP2D6* gene and results should be interpreted with caution, as hybrid alleles may cause inaccurate or misleading copy number estimates. If no variant alleles were detected, the allele was considered with normal metabolic activity (*CYP2D6Norm*).

**Table S1:** STROBE checklist

| Title and Abstract | |
| --- | --- |
| Item No: 1a | *Indicate the study’s design with Title and abstract 1 a commonly used term in the title or the abstract* |
| **In: Title.** | |
| Item No: 1b | *Provide in the abstract an informative and balanced summary of what was done*  *and what was found* |
| **In: Abstract; Sentences 4-8.** | |
| Introduction | |
| Item No: 2 | *Explain the scientific background and rationale for the investigation being reported* |
| **In: Introduction → Paragraph 1; Sentence 4.**  **In: Introduction → Paragraph 2; Sentence 8.** | |
| Item No: 3 | *State specific objectives, including any prespecified hypotheses* |
| **In: Introduction → Paragraph 3; Sentence 3.** | |
| Methods | |
| Item No: 4 | *Present key elements of study design early in the paper* |
| **In: Material and Methods → Paragraph 1, Sentence 2.** | |
| Item No: 5 | *Describe the setting, locations, and relevant dates, including periods of recruitment, exposure, follow-up, and data collection* |
| **In: Material and Methods → Paragraph 1, Sentence 1.**  **In: Material and Methods → Study participants and exposures, Sentence 4.** | |
| Item No: 6a | *Give the eligibility criteria, and the sources and methods of selection of participants. Describe methods of follow-up* |
| **In: Methods → Study participants and exposures; Sentences 1-3.** | |
| Item No: 6b | *For matched studies, give matching criteria and number of exposed and*  *Unexposed* |
| **N/A** | |
| Item No: 7 | *Clearly define all outcomes, exposures, predictors, potential confounders, and effect modifiers. Give diagnostic criteria, if applicable* |
| **In: Material and Methods → Clinical measurements and study outcomes; Paragraph 3; Sentences 3-4.**  **In: Material and Methods → Clinical measurements and study outcomes; Paragraph 2; Sentence 1.**  **In: Material and Methods → Statistical evaluation; Paragraph1; Sentence 8.**  **In: Material and Methods → Study participants and exposures; Sentence 2.** | |

(continued) **Table S1:** STROBE checklist

| Item No: 8 | *For each variable of interest, give sources of data and details of methods of assessment (measurement). Describe comparability of assessment methods if there is more than one group* |
| --- | --- |
| **In: Material and Methods → Clinical measurements and study outcomes; Paragraph 1; Sentences 5-8.**  **In: Material and Methods → Clinical measurements and study outcomes; Paragraph 2; Sentences 5-6.** | |
| Item No: 9 | *Describe any efforts to address potential sources of bias* |
| **In: Material and Methods → Study participants and exposures; Sentence 6.** | |
| Item No: 10 | *Explain how the study size was arrived at* |
| **In: Material and Methods → Statistical evaluation; Paragraph 1, Sentence 2.** | |
| Item No: 11 | *Explain how quantitative variables were handled in the analyses. If applicable, describe which groupings were chosen and why* |
| **In: Material and Methods → Clinical measurements and study outcomes; Paragraph 1; Sentence 7.** | |
| Item No: 12a | *Describe all statistical methods, including those used to control for confounding* |
| **In: Material and Methods → Statistical evaluation; Paragraph 1, Sentences 4-11.** | |
| Item No: 12b | *Describe any methods used to examine subgroups and interactions* |
| **In: Material and Methods → Statistical evaluation; Paragraph 1; Sentence 8.** | |
| Item No: 12c | *Explain how missing data were addressed* |
| **In: Results → Patient baseline characteristics; Sentence 1.** | |
| Item No: 12d | *If applicable, explain how loss to follow-up was addressed* |
| **In: Results → Patient baseline characteristics; Sentence 1.** | |
| Item No: 12e | *Describe any sensitivity analyses* |
| **In: Material and Methods → Statistical evaluations; Paragraph 3-4.** | |
| Results | |
| Item No: 13a | *Report numbers of individuals at each stage of study—eg numbers potentially eligible, examined for eligibility, confirmed eligible, included in the study, completing follow-up, and analysed* |
| **In: Results → Patient baseline characteristics; Sentence 1.** | |
| Item No: 13b | *Give reasons for non-participation at each stage* |
| **In: Results → Patient baseline characteristics; Sentence 1.** | |
| Item No: 13c | *Consider use of a flow diagram* |
| **N/A** | |
| Item No: 14a | *Give characteristics of study participants (eg demographic, clinical, social) and information on exposures and potential confounders* |
| **In: Results → Patient baseline characteristics; Sentences 2-7.** | |

(continued) **Table S1:** STROBE checklist

| Item No: 14b | *Indicate number of participants with missing data for each variable of interest* |
| --- | --- |
| **In: Results → Patient baseline characteristics; Sentences 1-2.** | |
| Item No: 14c | *Summarise follow-up time (eg, average and total amount)* |
| **In: Table 1.** | |
| Item No: 15 | *Report numbers of outcome events or summary measures over time* |
| **In: Table 2.**  **In: Results → Association between *CYP2D6* genotype and treatment effectiveness and tolerability; Sentence 2-4.**  **In: Figure 2.** | |
| Item No: 16a | Give unadjusted estimates and, if applicable, confounder-adjusted estimates and their precision (eg, 95% confidence interval). Make clear which confounders were adjusted for and why they were included |
| **In: Figure 1; Figure legend.** | |
| Item No: 16b | *Report category boundaries when continuous variables were categorized* |
| **In: Figure 1; Figure legend.** | |
| Item No: 16c | *If relevant, consider translating estimates of relative risk into absolute risk for a meaningful time period* |
| **N/A** | |
| Item No: 17 | *Report other analyses done—eg analyses of subgroups and interactions, and sensitivity analyses* |
| **In: Results → Sensitivity analysis; Sentence 1.** | |
| Discussion | |
| Item No: 18 | *Summarise key results with reference to study objectives* |
| **In: Discussion → Paragraph 1; Sentences 1-2.** | |
| Item No: 19 | *Discuss limitations of the study, taking into account sources of potential bias or imprecision. Discuss both direction and magnitude of any potential bias* |
| **In: Discussion → Limitations; Sentences 1-6.** | |
| Item No: 20 | *Give a cautious overall interpretation of results considering objectives, limitations, multiplicity of analyses, results from similar studies, and other relevant evidence* |
| **In: Discussion → Paragraph 2; Sentence 1.**  **In: Discussion → Paragraph 4; Sentences 1-4.** | |

(continued) **Table S1:** STROBE checklist

| Item No: 21 | *Discuss the generalisability (external validity) of the study results* |
| --- | --- |
| **In: Discussion → Paragraph 1; Sentence 3.**  **In: Discussion → Paragraph 2; Sentence 4.** | |
| Other information | |
| Item No: 22 | *Give the source of funding and the role of the funders for the present study and, if applicable, for the original study on which the present article is based* |
| **In: Funding information; Author Contributions.** | |

**Table S2:** Pharmacological treatment in patients with different CYP2D6 metabolizer status

|  | | CYP2D6-mediated metabolism | CYP2D6 inhibitory potency | Total | NM | IM | PM |
| --- | --- | --- | --- | --- | --- | --- | --- |
|  | |  |  | *n=*99 | *n=*73 | *n=*21 | *n=*5 |
| Total antidepressant dose  *(Fluoxetine equivalent mg)* | |  |  | 32  (19–44) | 32  (17–41) | 28  (19–45) | 40  (19–51) |
| **Antidepressant*** | Mirtazapine | Minor ^[61]^ |  | 34 | 26 | 5 | 3 |
|  | Escitalopram | Minor ^[59]^ | Weak ^[59]^ | 22 | 17 | 5 | 0 |
|  | Trazodone | Minor ^[60]^ |  | 20 | 15 | 4 | 1 |
|  | Mianserin | Moderate ^[62]^ |  | 14 | 8 | 6 | 0 |
|  | Venlafaxine | Major ^[59]^ |  | 12 | 9 | 2 | 1 |
|  | Sertraline | Minor ^[60]^ | Weak ^[59]^ | 11 | 9 | 1 | 1 |
|  | Clomipramine | Moderate ^[59]^ | Weak ^[59]^ | 7 | 4 | 2 | 1 |
|  | Tianeptine | No |  | 6 | 5 | 1 | 0 |
|  | Fluoxetine | Moderate ^[59]^ | Strong ^[59]^ | 4 | 4 | 0 | 0 |
|  | Citalopram | Minor ^[59]^ | Weak ^[59]^ | 4 | 2 | 2 | 0 |
|  | Duloxetine | Moderate ^[60]^ | Moderate ^[59]^ | 4 | 4 | 0 | 0 |
|  | Paroxetine | Moderate ^[60]^ | Strong ^[59]^ | 3 | 2 | 1 | 0 |
|  | Maprotiline | Minor ^[63]^ |  | 2 | 1 | 1 | 0 |
|  | Bupropion | No | Strong ^[59]^ | 1 | 1 | 0 | 0 |
|  |  |  |  |  |  |  |  |

The table summarizes drugs identified as CYP2D6 substrates, along with their sources. References correspond to the numbering in the main manuscript. Data presented as absolute number.

*Some patients used more than one antidepressant during the study period.

*NM: Normal CYP2D6 metabolizer; IM: Intermediate CYP2D6 metabolizer; PM: Poor CYP2D6 metabolizer.*

**Table S3:** Sensitivity analysis: Influence of the exclusion of the patients not treated with CYP2D6 substrate drug on the primary efficacy outcomes

| Outcome | Original analysis  *PM n = 5; IM n = 21; NM n = 73* | Only CYP2D6 substrates  *PM n = 5; IM n = 20; NM n = 68* |
| --- | --- | --- |
| **HAMD % change from baseline at V1** | | |
| PM vs NM  *Difference of means [CI95%]* | 16% [0.9%; 31%]  *n=99, p=0.033* | 16% [0.6%; 31%]  *n=93, p=0.040* |
| IM vs NM  *Difference of means [CI95%]* | 11% [3%; 19%;]  *n=99, p=0.006* | 11% [2.1%; 19%]  *n=93, p=0.009* |
| **HAMD % change from baseline at V2** | | |
| PM vs NM  *Difference of means [CI95%]* | 32% [17%; 48%]  *n=99, p<0.001* | 33% [18%; 49%;]  *n=93, p<0.001* |
| IM vs NM  *Difference of means [CI95%]* | 19% [11%; 28%]  *n=99, p<0.001* | 20% [11%; 28%]  *n=93, p<0.001* |
| **BDI-II % change from baseline at V1** | | |
| PM vs NM  *Difference of means [CI95%]* | 9% [-12%; 31%]  *n=99, p > 0.1* | 10% [-12%; 32%]  *n=93, p > 0.1* |
| IM vs NM  *Difference of means [CI95%]* | 12% [0.4%; 23%]  *n=99, p=0.041* | 13% [0.7%; 25%]  *n=93, p=0.035* |
| **BDI-II %change from baseline at V2** | | |
| PM vs NM  *Difference of means [CI95%]* | 26% [7%; 44%]  *n=99, p=0.004* | 26% [7%; 46%;]  *n=93, p=0.004* |
| IM vs NM  *Difference of means [CI95%]* | 19% [9%; 29%]  *n=99, p<0.001* | 20% [9%; 30%]  *n=93, p<0.001* |
| **Response rate at V2** | | |
| PM vs NM  *Difference in response rates* | 61%  *n=99, p<0.001* | 61%  *n=93, p < 0.001* |
| IM vs NM  *Difference in response rates* | 52%  *n=99, p<0.024* | 51%  *n=93, p=0.021* |

Exclusion of the individuals not treated with the drugs considered as CYP2D6 substrates does not significantly change the magnitude or the significance levels of the between-group differences in the efficacy outcomes. *V1 - 2 weeks after the admission; V2 - 4-6 weeks after the hospital admission.*

**Table S4:** Sensitivity analysis: Influence of the exclusion of patients not treated with CYP2D6 substrate drug on the primary tolerability outcomes

| Outcome | Original analysis  *PM n = 5; IM n = 21; NM n = 73* | Only CYP2D6 substrates  *PM n = 5; IM n = 20; NM n = 68* |  |
| --- | --- | --- | --- |
| **Average side-effect intensity at V2** | | | |
| PM Median [IQR] vs  NM Median [IQR] | 4.6 [3.3; 5.3], 2.3 [1.8; 3.1]  *n=99, p=0.002* | 4.7 [3.3; 5.3], 2.3 [1.8; 3.1]  *n=93, p<0.001* |  |
| IM Median [IQR] vs  NM Median [IQR] | 3.6 [2.9; 4.1], 2.3 [1.8; 3.1]  *n=99, p<0.001* | 3.4 [2.9; 4.2], 2.3 [1.8; 3.1]  *n=93, p=0.001* |  |
| **CNS side-effect intensity at V2** | | | |
| PM Median [IQR] vs  NM Median [IQR] | 3.2 [2.6; 4.8], 1.9 [1.5; 2.3]  *n=99, p=0.002* | 3.2 [2.6; 4.8], 1.9 [1.5; 2.4]  *n=93, p=0.001* |  |
| IM Median [IQR] vs  NM Median [IQR] | 2.9 [2.0; 3.7], 1.9 [1.5;2.3]  *n=99, p=0.002* | 2.7 [2.0; 3.8], 1.9 [1.5; 2.4]  *n=93, p=0.001* |  |
| **GI side-effect intensity at V2** | | | |
| PM Median [IQR] vs  NM Median [IQR] | 2.9 [2.4; 3.4], 1.6 [1.3; 2.1]  *n=99, p=0.001* | 2.9 [2.4; 3.4], 1.6 [1.3; 2.1]  *n=93, p=0.002* |  |
| IM Median [IQR] vs  NM Median [IQR] | 2.3 [1.6; 2.9], 1.6 [1.3; 2.1]  *n=99, p=0.004* | 2.3 [1.6; 2.9], 1.6 [1.3; 2.1]  *n=93, p=0.002* |  |
| **SF side-effect intensity at V2** | | | |
| PM Median [IQR] vs  NM Median [IQR] | 9.0 [3.5; 9.0], 3.7 [2.7; 6.0]  *n=99, p=0.067* | 9.0 [3.5; 9.0], 3.7 [2.7; 6.0]  *n=93, p =0.091* |  |
| IM Median [IQR] vs  NM Median [IQR] | 6.0 [3.8; 9.0], 3.7 [2.7; 6.0]  *n=99, p>0.1* | 6.0 [3.8; 9.0], 3.7 [2.7; 6.0]  *n=93, p=0.047* |  |

After the exclusion of the individuals not treated with the drugs considered as CYP2D6 substrates, the magnitude of the between-group changes persisted.

*V1: 2 weeks after the admission; V2: 4-6 weeks after the hospital admission; CNS – Central nervous system, GI – Gastro-intestinal tract, SF – Sexual function.*

**Table S5:** Frequency of CYP2D6 × CYP2C19 combination phenotypes in the cohort.

|  |  | CYP2C19 metabolizer group | | | |
| --- | --- | --- | --- | --- | --- |
|  |  | RM | NM | PM | Total |
| 2D6 metabolizer group | NM | 28 | 36 | 9 | 73 |
|  | IM | 7 | 4 | 10 | 21 |
|  | PM | 1 | 1 | 3 | 5 |
|  | Total | 36 | 41 | 22 | 99 |

Data presented as absolute number.

*RM: Rapid metabolizer; NM: Normal metabolizer; IM: Intermediate metabolizer; PM: Poor metabolizer.*

**Table S6:** Comparison of treatment outcomes and adverse effect burden by CYP2D6/CYP2C19 metabolic capacity.

|  | Normal Metabolizers (CYP2D6 NM + CYP2C19 NM/RM)  n=64 | Reduced on One Enzyme (CYP2D6 or CYP2C19)  n=22 | Reduced on Both Enzymes (CYP2D6 and CYP2C19)  n=13 |
| --- | --- | --- | --- |
| **Absolute HAMD score reduction (Mean ± SD)** | −19 ± 5.2 | −15 ± 6.0 | −11 ± 5.4 |
| Difference vs NM | Reference | 4.0 [95% CI: 0.7-7.2] **p = 0.01** | 7.7 [95% CI: 3.7-12] **p <0.001** |
| **Absolute BDI-IA score reduction (Mean ± SD)** | −21 ± 9.9 | −19 ± 8.8 | −13 ± 10 |
| Difference vs NM | Reference | 2.1 [95% CI: -7.9-3.7] p > 0.1 | 7.8[95% CI: 0.7-15] **p = 0.026** |
| **Relative HAMD score reduction (Mean ± SD)** | −61% ± 11 | −44% ± 16 | −31% ± 15 |
| Difference vs NM | Reference | 17 [95% CI: 9-25] **p < 0.001** | 30 [95% CI: 21-40] **p < 0.001** |
| **Relative BDI-IA score reduction (Mean ± SD)** | −60% ± 14 | −44% ± 17 | −31% ± 21 |
| Difference vs NM | Reference | 16 [95% CI: 6-26] **p < 0.001** | 30 [95% CI: 18-41] **p < 0.001** |
| **Response rate (≥50% HAMD reduction)** | 86% (55/64) | 46% (10/22) | 7.7% (1/13) |
| Difference vs NM | Reference | 40 [95% CI: 18-63] **p < 0.003** | 78 [95% CI: 61-95] p = 0.065 |
| **Response rate (≥50% BDI-IA reduction)** | 86% (55/64) | 36% (8/22) | 7.7% (1/13) |
| Difference vs NM | Reference | 50 [95% CI: 28-72] **p < 0.001** | 79 [95% CI: 64-95] p = 0.094 |
| **Average TSES Intensity score (All AEs) - median [IQR]** | 2.3 [1.8–3.1] | 3.1 [2.1–4.0] | 3.6 [3.1–4.3] |
| Difference vs NM | Reference | 0.8; p > 0.1 | 1.3; **p < 0.001** |
| **Average TSES Intensity score (CNS AEs) - median [IQR]** | 1.7 [1.5–2.1] | 2.6 [1.9–3.1] | 3.2 [2.4–3.8] |
| Difference vs NM | Reference | 0.6; p > 0.1 | 1.5; **p < 0.001** |
| **Average TSES Intensity score (GI AEs) - median [IQR]** | 1.6 [1.3–2.1] | 1.8 [1.5–2.6] | 2.3 [2.1–3.6] |
| Difference vs NM | Reference | 0.2; **p = 0.029** | 0.7; **p < 0.001** |
| **Average TSES Intensity score (SF AEs) - median [IQR]** | 3.7 [2.7–6.0] | 6.0 [3.7–7.3] | 6.0 [3.7–9.0] |
| Difference vs NM | Reference | 2.3; p > 0.1 | 2.3; p = 0.061 |

Patients were divided into three groups based on their *CYP2D6* and *CYP2C19* genotype. Treatment outcomes were poorest in the group with reduced function in both enzymes. In patients with reduced function of one enzyme, the results were also worse compared to NM. Adverse effects followed the same pattern.

**Table S7:** Sensitivity analysis: Influence of CYP2D6 metabolic capacity (alternative classification) on primary treatment outcomes and adverse effect burden

|  | NM  (*1/*1; *1/*2)  n=64 | IM  (*1/*3; *1/*4; *1/*41; *1/*5; *1/*6; ; 1/*9; *2/*41; *4/*41; *5/*9)  n=30 | PM  (*3/*3; *4/*4)  n=5 |
| --- | --- | --- | --- |
| **Absolute HAMD score reduction (Mean ± SD)** | −19 ± 5.1 | −14 ± 5.1 | −9.2 ± 9.5 |
| Difference vs NM | Reference | 5.1 [95% CI: 2.3-8.0] **p < 0.001** | 9.5 [95% CI: 3.5.-16.0] **p = 0.001** |
| **Absolute BDI-IA score reduction (Mean ± SD)** | −21 ± 9.0 | −17 ± 11 | −13 ± 8.2 |
| Difference vs NM | Reference | 4.6 [95% CI: 4.1.-13] **p < 0.001** | 7.5 [95% CI: -3.6-17] p > 0.1 |
| **Relative HAMD score reduction (Mean ± SD)** | −60% ± 12 | −43% ± 16 | −24% ± 23 |
| Difference vs NM | Reference | 17 [95% CI: 9.9-25] **p < 0.001** | 34 [95% CI: 18-49] **p < 0.001** |
| **Relative BDI-IA score reduction (Mean ± SD)** | −60% ± 13 | −40% ± 21 | −31% ± 15 |
| Difference vs NM | Reference | 19 [95% CI: 11-28] **p < 0.001** | 28 [95% CI: 9.2-46] **p = 0.001** |
| **Response rate (≥50% HAMD reduction)** | 84% (54/64) | 37% (11/30) | 20% (1/5) |
| Difference vs NM | Reference | 48 [95% CI: 28-66] **p < 0.001** | 64 [95% CI: 28-100] **p = 0.016** |
| **Response rate (≥50% BDI-IA reduction)** | 84% (54/64) | 33% (10/30) | 0% (0/5) |
| Difference vs NM | Reference | 51 [95% CI: 30-67] **p < 0.001** | 84 [95% CI: 40-91] **p = 0.001** |
| **Average TSES Intensity score (All AEs) - median [IQR]** | 2.3 [1.8–3.1] | 3.1 [2.1–3.9] | 4.6 [3.3–5.3] |
| Difference vs NM | Reference | 0.8; **p = 0.007** | 2.3; **p = 0.002** |
| **Average TSES Intensity score (CNS AEs) - median [IQR]** | 1.9 [1.5–2.2] | 2.5 [1.8–3.2] | 3.2 [2.6–4.8] |
| Difference vs NM | Reference | 0.6; **p = 0.001** | 1.3; **p = 0.004** |
| **Average TSES Intensity score (GI AEs) - median [IQR]** | 1.6 [1.3–2.1] | 2.0 [1.5–2.8] | 2.9 [2.4–3.4] |
| Difference vs NM | Reference | 0.4; **p = 0.008** | 1.3; **p = 0.002** |
| **Average TSES Intensity score (SF AEs) - median [IQR]** | 3.8 [2.7–6.0] | 5.7 [3.7–7.8] | 9.0 [3.5–9.0] |
| Difference vs NM | Reference | 1.9; p = 0.058 | 5.2; p = 0.093 |

Patients were categorized into CYP2D6 metaboliser groups according to alternative classification [42]. Key treatment outcomes and adverse effect burden were compared across groups. Both IMs and PMs showed poorer antidepressant response and higher adverse effect intensity compared with NMs.

*NM: Normal metabolizer; IM: Intermediate metabolizer; PM: Poor metabolizer.*

**Table S8:** Sensitivity analysis: Influence of CYP2D6 phenoconversion-adjusted phenotype reclassification on primary treatment outcomes and adverse effect burden

|  | NM  n=71 | IM  n=19 | PM  n=9 |
| --- | --- | --- | --- |
| **Absolute HAMD score reduction (Mean ± SD)** | −18 ± 5.3 | −14 ± 5.7 | −13 ± 7.9 |
| Difference vs NM | Reference | 4.9 [95% CI: 1.3–8.5] **p = 0.004** | 4.8 [95% CI: -0.–9.7] p = 0.058 |
| **Absolute BDI-IA score reduction (Mean ± SD)** | −21 ± 9.8 | −16 ± 9.5 | −18± 9.9 |
| Difference vs NM | Reference | 5.0 [95% CI: -13-11] p > 0.1 | 0.9. [95% CI: -7.7-957] p > 0.1 |
| **Relative HAMD score reduction (Mean ± SD)** | −59% ± 12 | −38% ± 17 | −37% ± 23 |
| Difference vs NM | Reference | 20 [95% CI: 11-30] **p < 0.001** | 19 [95% CI: 6.0-32] **p = 0.001** |
| **Relative BDI-IA score reduction (Mean ± SD)** | −58% ± 16 | −37% ± 21 | −43% ± 19 |
| Difference vs NM | Reference | 20 [95% CI: 9-31] **p < 0.001** | 13 [95% CI: -2.1-28] p > 0.1 |
| **Response rate (≥50% HAMD reduction)** | 80% (57/71) | 26% (5/19) | 44% (4/9) |
| Difference vs NM | Reference | 54 [95% CI: 29-70] **p < 0.001** | 36 [95% CI: 5-63] **p = 0.0027** |
| **Response rate (≥50% BDI-IA reduction)** | 79% (56/71) | 26% (5/19) | 33% (3/9) |
| Difference vs NM | Reference | 53 [95% CI: 28-69] **p = 0.001** | 46 [95% CI: 10-71] **p = 0.009** |
| **Average TSES Intensity score (All AEs) - median [IQR]** | 2.3 [1.8–3.1] | 3.7 [2.8–4.9] | 3.2 [2.8–4.9] |
| Difference vs NM | Reference | 1.4; **p = 0.001** | 0.9; **p = 0.005** |
| **Average TSES Intensity score (CNS AEs) - median [IQR]** | 1.9 [1.5–2.4] | 2.9 [2.1–3.8] | 2.5 [1.9–3.7] |
| Difference vs NM | Reference | 1.0; **p < 0.001** | 0.6; **p = 0.009** |
| **Average TSES Intensity score (GI AEs) - median [IQR]** | 1.8 [1.3–2.1] | 2.3 [1.5–2.9] | 2.4 [1.4–3.1] |
| Difference vs NM | Reference | 0.5; **p = 0.007** | 0.6; p > 0.1 |
| **Average TSES Intensity score (SF AEs) - median [IQR]** | 3.7 [2.7–6.0] | 6.0 [3.7–9.0] | 6.0 [4.2–9.0] |
| Difference vs NM | Reference | 2.3; **p = 0.004** | 2.3; **p = 0.044** |

Patients were reclassified into CYP2D6 metaboliser groups after their phenotype was adjusted for drug-induced phenoconversion. Treatment outcomes and adverse effect burden were compared across the reclassified groups. Individuals with phenoconversion-adjusted IM and PM status showed significantly lower antidepressant response rates and a higher intensity of adverse effects compared with NMs.

*NM: Normal metabolizer; IM: Intermediate metabolizer; PM: Poor metabolizer.*

**Figure S1:** Sensitivity analysis: Association between antidepressant treatment effectiveness and *CYP2D6* genotype in patients receiving CYP2D6-substrate antidepressants

Treatment effectiveness outcomes are presented only for patients treated with CYP2D6-substrate antidepressants. The results are consistent with those in the full cohort, with no significant changes in response or remission rates.

*HAMD: Hamilton Depression Rating Scale; BDI-IA: Beck depression inventory; NM: Normal CYP2D6 metabolizer; IM – Intermediate metabolizers; PM: Poor CYP2D6 metabolizer. *p<0.05, ** p<0.01, *** p<0.001, whiskers represent CI 95%*

**Figure S2:** Sensitivity analysis: Association between antidepressant treatment tolerability and *CYP2D6* genotype in patients receiving CYP2D6-substrate antidepressants

Tolerability outcomes are shown only for patients receiving CYP2D6-substrate antidepressants. The findings are similar to those in the full cohort, with no significant differences in adverse event rates.

*CNS - central nervous system; GI - gastrointestinal; SF - sexual function; TSES – Toronto Side-Effects Scale,*p<0.05, ** p<0.01, *** p<0.001. Boxes represent interquartile range and dots represent outliers.*

**Figure S3:** Sensitivity analysis: Association between antidepressant treatment effectiveness and *CYP2D6* genotype, with patients classified according to alternative classification

Figure displays the main antidepressant effectiveness outcomes across CYP2D6 metabolizer groups defined by alternative classification. The results are practically unchanged when patients are categorized in this way.

*HAMD: Hamilton Depression Rating Scale; BDI-IA: Beck depression inventory; NM: Normal CYP2D6 metabolizer; IM – Intermediate metabolizers; PM: Poor CYP2D6 metabolizer. *p<0.05, ** p<0.01, *** p<0.001, whiskers represent CI 95%*

**Figure S4:** Sensitivity analysis: Association between antidepressant treatment tolerability and *CYP2D6* genotype, with patients classified according to alternative classification

Tolerability outcomes are shown for patients categorized by CYP2D6 metabolizer status according to alternative classification. The findings are similar to those in the full cohort, with no meaningful differences in adverse event intensity.

*CNS - central nervous system; GI - gastrointestinal; SF - sexual function; TSES – Toronto Side-Effects Scale,*p<0.05, ** p<0.01, *** p<0.001. Boxes represent interquartile range and dots represent outliers.*

**Figure S5:** Sensitivity analysis: Association between antidepressant treatment effectiveness and CYP2D6 phenotype after phenoconversion-adjusted reclassification

Figure displays the main antidepressant effectiveness outcomes across CYP2D6 metabolizer groups after adjusting phenotypes for drug-induced phenoconversion. The results remain consistent,

*HAMD: Hamilton Depression Rating Scale; BDI-IA: Beck depression inventory; NM: Normal CYP2D6 metabolizer; IM – Intermediate metabolizers; PM: Poor CYP2D6 metabolizer, *p<0.05, ** p<0.01, *** p<0.001, whiskers represent CI 95%.*

**Figure S6:** Sensitivity analysis: Association between antidepressant treatment tolerability and CYP2D6 phenotype after phenoconversion-adjusted reclassification

Tolerability outcomes are shown for patients categorized by CYP2D6 metabolizer status after phenoconversion-adjusted reclassification. The findings are consistent with those in the full cohort, with no meaningful differences in adverse event intensity.

*CNS - central nervous system; GI - gastrointestinal; SF - sexual function; TSES – Toronto Side-Effects Scale,*p<0.05, ** p<0.01, *** p<0.001. Boxes represent interquartile range and dots represent outliers.*

**References:**

59. https://drug-interactions.medicine.iu.edu/main-table (accessed 10 Nov2025).

60. ClinPGx. ClinPGx. https://www.clinpgx.org/ (accessed 10 Nov2025).

61. Lind A-B, Reis M, Bengtsson F, Jonzier-Perey M, Powell Golay K, Ahlner J et al. Steady-state concentrations of mirtazapine, N-desmethylmirtazapine, 8-hydroxymirtazapine and their enantiomers in relation to cytochrome P450 2D6 genotype, age and smoking behaviour. Clin Pharmacokinet 2009; 48: 63–70.

62. Hole K, Molden E. Association Between CYP2D6 Genotypes and Serum Concentrations of Mirtazapine and Mianserin. Basic Clin Pharmacol Toxicol 2025; 136: e70013.

63. Brachtendorf L, Jetter A, Beckurts KT, Hölscher AH, Fuhr U. Cytochrome P450 enzymes contributing to demethylation of maprotiline in man. Pharmacol Toxicol 2002; 90: 144–149
